# Sensitized Radiation-Induced Polymerization of Indene with 1,1,2,2-Tetrachloroethane

**DOI:** 10.3390/polym17111550

**Published:** 2025-06-02

**Authors:** Ransel Barzaga, Domingo Aníbal García-Hernández, Arturo Manchado, Ilaria Di Sarcina, Alessia Cemmi, Franco Cataldo

**Affiliations:** 1 Instituto de Astrofísica de Canarias (IAC), Vía Láctea s/n, E-38205 La Laguna, Spain; ransel.barzaga@iac.es (R.B.); anibal.garcia.hernandez@iac.es (D.A.G.-H.); amt@iac.es (A.M.); 2 Departamento de Astrofísica, Universidad de La Laguna (ULL), E-38206 La Laguna, Spain; 3 Consejo Superior de Investigaciones Científicas, 28006 Madrid, Spain; 4 ENEA Nuclear Department, Casaccia R.C., 00123 Rome, Italy; ilaria.disarcina@enea.it (I.D.S.); alessia.cemmi@enea.it (A.C.); 5 Actinium Chemical Research Institute, Via Casilina 1626a, 00133 Rome, Italy

**Keywords:** indene, radiation-induced polymerization, sensitizer, 1,1,2,2-tetrachloroethane, polyindene, kinetics, mechanism, molecular weight, gamma radiation

## Abstract

Sensitized radiation-induced polymerization of indene monomer was achieved at a dose rate of 3 kGy/h. The sensitizer (1,1,2,2-tetrachloroethane or TCE) leads to higher polyindene yields and faster polymerization kinetics with respect to bulk radiation-induced polymerization of indene. The radiation chemical yield G_p_ was found to increase with the dose in sensitized polymerization of indene following a power law, while an opposite trend was detected in the absence of the sensitizer. The sensitizer enhances the cationic polymerization mechanism in parallel to the free radical mechanism, as shown with both electronic absorption spectroscopy and FT–IR analysis of the polyindenes. Despite the enhancement of the polymer yield and the faster polymerization kinetics offered by the presence of TCE, the molecular weight of the resulting polyindene was found to be rather low. This was true whether the molecular weight was measured by end group analysis using X-ray fluorescence or the glass transition temperature determination with respect to the polyindenes produced with γ radiation without the sensitizer or with a pure cationic mechanism.

## 1. Introduction

Our current works on radiation-induced polymerization of indene [1,2] are due to the recent and intriguing discovery of indene and cyanoindene in space, specifically in the Taurus Molecular Cloud (TMC-1) [3,4,5]. These discoveries have also stimulated our researches into the reaction products of indene with fullerene [6,7]. Other authors have focused on the reactivity of indene with the ice mantle in the interstellar medium under the action of high energy photons [8]. From the above astrochemical perspective, it is interesting to know how indene behaves toward high energy radiation fields present in the interstellar medium and represented by cosmic rays, γ-rays, X-rays and UV photons. In the previous work, we showed that indene is prone to polymerize in bulk to polyindene when subjected to γ irradiation at different dose rates [1]. This implies that polymeric indene may also be present in the interstellar medium [1]. Furthermore, the chemical structure of radiation-polymerized indene was studied in comparison to the structure of polyindene produced by chemical initiation [2]. In the future, it will be interesting to combine our experimental results with the use of the new techniques of machine learning to simulate the destiny of indene in a harsh space environment [9].

It was shown that the γ-ray-induced polymerization of indene in bulk involves a mixed free radical and ionic mechanism, with the neat prevalence of the former over the latter [1]. In the present work, indene was irradiated in presence of a chlorinated solvent, i.e., 1,1′,2,2′-tetrachloroethane, which should act as a sensitizer affecting the polymerization mechanism and moreover the polymer yield. The kinetics, mechanism and final structure of the resulting polyindene in this sensitized radiation-induced polymerization is the object of the present study.

## 2. Materials and Methods

### 2.1. Materials and Equipment

Indene was supplied by Merck (Darmstadt, Germany) and distilled immediately prior to use, removing the phenolic stabilizer and leaving out the tail as distillation residue. All the other solvents and reagents were obtained from Merck and used as received.

The spectrophotometric analysis of the irradiated indene solutions was made with a Shimadzu UV2450 (Kyoto, Japan).

The thermogravimetric analysis (TGA) with simultaneous Differential Scanning Calorimetric analysis (DSC) was made by a Mettler-Toledo (Greifensee, Switzerland) model TGA/DSC-3^+^ Star System at a heating rate of 20 °C/min, using alumina crucibles and N_2_ flow at 50 mL/min.

The DSC analysis was performed at a heating rate of 10 °C/min on a Mettler (Greifensee, Switzerland) DSC-1 using aluminum crucibles with punched caps under N_2_ flow of 10 mL/min.

The FT–IR spectra were collected on a Nicolet iS50 spectrometer from Thermo-Fisher Scientific (Waltham, MA, USA) in reflectance mode using the ATR Smart iTX accessory. Through the Omnic-9 software of the FT–IR spectrometer, the reflectance spectra were transformed into the most common transmittance/absorbance IR spectra.

The XRF (X-ray fluorescence) analysis on the polyindene samples was made on a Spectrocube model XEP06C spectrometer from Ametek (Berwyn, PA, USA, calibrated for light element measurements according to ASTM D7751. The XRF measurement was made directly on each polyindene sample. The samples were transferred in a purpose-designed sample holder (i.e., a polyethylene cup with a Chemplex polypropylene membrane).

The irradiation tests were carried out at the Calliope γ irradiation facility (ENEA Casaccia Research Center, Rome, Italy) [10], using a dose rate of 3.0 kGy/h. The samples absorbed doses up to 1000 kGy.

### 2.2. Irradiation Experiments

The freshly distilled indene (47.88 g) was mixed in a flask with 3.69 g of 1,1,2,2-tetrachloroethane (TCE), to produce a stock solution containing 95% by mol of indene and 5% by mol of TCE. The stock solution was then used to fill a series of Wheaton glass vials having an internal volume of 8.5 mL each. The stock solution contained in each vial was nitrogen flushed for at least two minutes with a capillary submerged into the flask, before proceeding with the sealing step using a black bakelite solid top cap with white styrene-butadiene rubber liner inside.

The flasks were then transferred into a glove bag (Aldrich AtmosBag type), supplied by Aldrich, Burlington, MA, USA, kept under nitrogen, and subjected to γ irradiation from a ^60^Co source at a dose rate of 3.0 kGy/h. Six vials were irradiated respectively at 50, 100, 200, 400, 700 and 1000 kGy. After irradiation, all of the samples were stored in the cold [10] and analyzed just a few days after the irradiation exposure.

### 2.3. Determination of Polymer Yield by Thermogravimetric Analysis (TGA-DTG)

A weighed amount of the selected radiolysed indene solution at a given dose was placed into an alumina crucible and subjected to thermogravimetric analysis at a heating rate of 20 °C/min. The analysis was performed from 25 °C to 600 °C under nitrogen flow. Above 600 °C and up to 900 °C, the gas was switched to an oxygen flow. The polyindene yield as well as the indene dimer/trimer yield, where applicable, was determined by the combined analysis of the TGA and DTG curves, a method already successfully applied in the previous study [1].

### 2.4. Determination of Polymer Yield by Polymer Precipitation and Gravimetry

After irradiation at a given dose, the selected vial was opened and its content weighed (typical weight 7.5 to 7.8 g). The content was poured into a flask containing 100 mL of anhydrous ethanol and magnetically stirred vigorously at ambient temperature. Stirring was prolonged until all the polymer was separated into whitish-orange flakes from the liquid phase. At this point, stirring was stopped and the polymer settled at the bottom of the flask. It was easily collected by decantation. The polymer was then washed (by stirring) in other 50 mL of anhydrous ethanol. The resulting slurry was quickly filtered in a Buchner filter and washed further with ethanol before being collected and left to dry first in air and then in a desiccator until it reached a constant weight. It was noticed that the indene solutions radiolysed at 1000 and at 700 kGy appeared initially orange-reddish in color and released the polymer slowly. Thus, long stirring time was necessary to isolate the polyindene. Furthermore, the orange color was quite persistent, although it gradually faded into yellow. On the other hand, all of the other indene solutions radiolysed at doses below 700 kGy appeared yellow in color from the beginning and quite promptly released the polymer as a whitish solid precipitate. The presence of free hydrochloric acid produced by the radiolysis of the indene-TCE mixture was detected with indicator paper either in the washing ethanolic solutions or in the precipitated polyindene.

## 3. Results and Discussion

### 3.1. Polymer Yield and Polymerization Kinetics

#### 3.1.1. Polyindene Yield Determination by Thermogravimetric and Gravimetric Analysis

**Figure 1:**
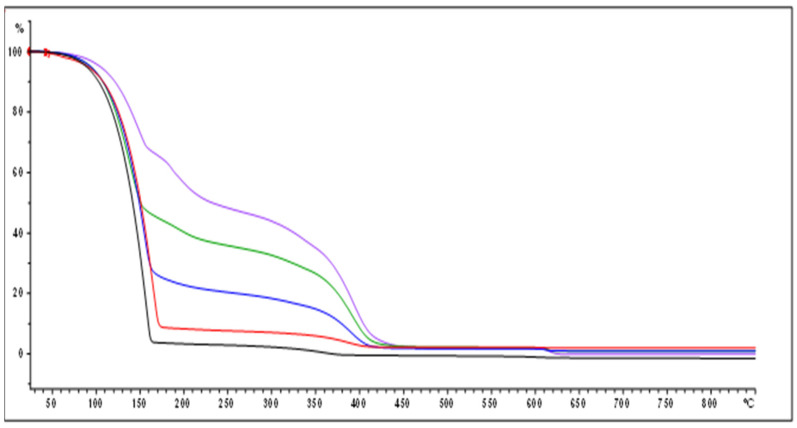
Thermogravimetric analysis (TGA) of radiolysed indene solutions at 1000 (violet), 700 (green), 400 (blue), 100 (red) and 50 (black) kGy absorbed dose. The analysis was made at a heating rate of 20 °C/min from 25 °C to 600 °C under N_2_ flow and then to 900 °C under O_2_ flow.

**Figure 2:**
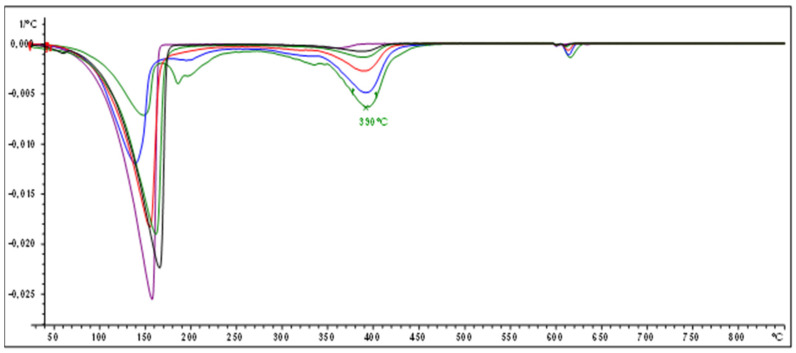
DTG (first derivative of the TGA shown in Figure 1) on radiolysed indene solutions. The peaks at about 155–165 °C are due to the vaporization of the monomer and TCE. The peak at about 390–400 °C is due to polyindene. At 600 °C, there is the burning step of the small amount of char formed during polyindene thermal decomposition. Color codes: 1000 (dark green),700 (blue), 400 (red), 200 (green), 100 (black) and 50 (violet) kGy absorbed dose.

**Figure 3:**
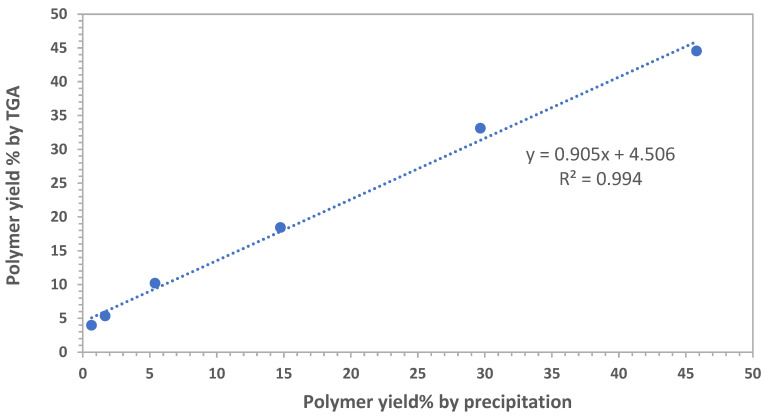
Comparison of polymer yield as determined by precipitation method versus the TGA method (see text for more details). The polymer yield increased with radiation dose. Thus, the highest yield was obtained at 1000 kGy and the other progressively lower yields respectively at the other doses used (i.e., 700, 400, 200, 100 and 50 kGy). See also Table 1.

In a previous work [1], it was shown that it is possible to determine the amount of polyindene produced by γ irradiation via a straight thermogravimetric analysis of the irradiated monomer containing the dissolved polymer. In fact, the irradiation of indene causes the formation of the polyindene, which is soluble in the monomer [1]. In the present study, a sensitizer, i.e., 1,1,2,2-tetrachloroethane (TCE), was added to the monomer as detailed in the experimental section. TCE is also a solvent of polyindene. Thus, after γ irradiation at different doses (i.e., 50, 100, 200, 400, 700 and 1000 kGy) the resulting homogeneous solutions were subjected to thermogravimetric analysis (TGA) as shown in Figure 1. The latter figure should be interpreted in conjunction with the corresponding DTG curves (first derivative of TGA) of Figure 2. Figure 1 shows that the indene monomer and TCE are almost completely evaporated at about 155–165 °C, leaving a polymer residue which decomposes at about 400 °C. The residual char is burned with oxygen above 600 °C. From the first glance at Figure 1, it is immediately evident that the polyindene yield was dependent on the dose and is maximized at 1000 kGy, as expected. By combining the TGA and DTG data (Figure 1 and Figure 2) the polyindene yield was determined, and it is shown in Figure 3 in comparison with the polyindene yield as determined by precipitation (see next section).

#### 3.1.2. Determination of Polyindene Yield

An alternative and traditional way to determine the polymer yield involves the precipitation of the polymer from its radiolysed solution using a non-solvent. As detailed in the experimental section, the irradiated indene solutions were treated with a large excess of anhydrous ethanol to cause the polyindene precipitation, collection by filtration and its gravimetric determination after washing and drying.

In Figure 3, the polyindene yields determined by precipitation and gravimetry are compared with the polymer yields measured by TGA. The accord between the two method appears linear and satisfactory:

(Polyindene yield by TGA) = 0.905 (Polyindene yield by precipitation) + 4.(1)

However, the above correlation equation suggests that the TGA method gives an overestimation of the yield as truly determined by the precipitation method.

The offset of the intercept at 4.5% confirms that, especially at low conversions (low absorbed dose), the TGA overestimation of the polyindene yield is larger, becoming smaller at higher conversions and dose (see Table 1).

#### 3.1.3. Kinetics of Indene Polymerization in Presence of TCE Sensitizer

**Figure 4:**
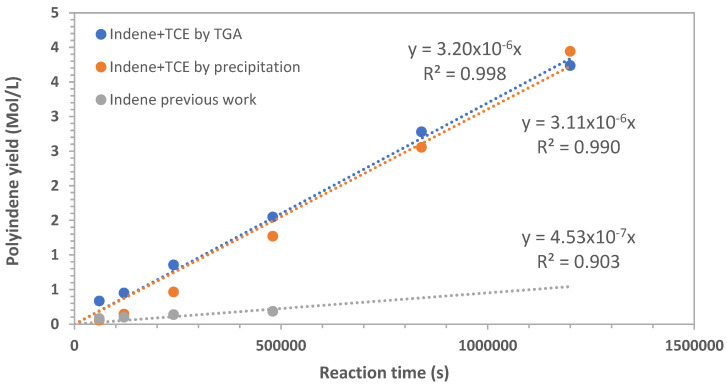
The rate constant of indene polymerization at 3 kGy/h in the presence of 5% mol of TCE sensitizer is found at 3.11 × 10^−6^ mol L^−1^ s^−1^ using the gravimetric data of polyindene precipitation; the TGA data suggest an indene conversion rate constant of 3.20 × 10^−6^ mol L^−1^ s^−1^; the averaged rate constant of bulk indene without sensitizers is shown at 4.53 × 10^−7^ mol L^−1^ s^−1^ (for a dose rate of 3 kGy/h).

In a previous work [1], indene monomer was irradiated in bulk at different dose rates. At 2 kGy/h, the indene polymerization kinetics rate constant was found at 3.68 × 10^−7^ mol L^−1^ s^−1^, while at 4 kGy/h the kinetics rate constant was found at 5.38 × 10^−7^ mol L^−1^ s^−1^. These two rate constants were averaged at 4.53 × 10^−7^ mol L^−1^ s^−1^ (as shown in Figure 4) to be compared with the kinetic rate constant measured on indene polymerization at 3 kGy/h when sensitized by the presence of TCE (5% mol). As expected, the presence of TCE enhanced the polymerization rate constant of indene by an order of magnitude, i.e., k_p_ = 3.11 × 10^−6^ mol L^−1^ s^−1^ if the gravimetric data of polyindene yields are used (see Figure 4). As explained in the previous section, the TGA analysis of the irradiated indene solutions gave an overestimation of the polyindene yield. Thus, using the TGA data, the rate constant of indene conversion appears enhanced by a factor of 1.03, leading to a k_p_ value of 3.20 × 10^−6^ mol L^−1^ s^−1^. At the end, the deviation in the polymerization rate constant introduced by the TGA measurements is rather small. As reviewed by Ivanov [11], the addition of a sensitizer (in the form of a chlorinated hydrocarbon) to a monomer leads to an enhancement of the polymerization rate and to a significant increase in the polymer yield. The latter point will be more deeply examined in the next section in the discussion of the radiation chemical yield G_p_ tool.

### 3.2. Radiation Chemical Yield in Sensitized Radiation-Induced Polymerization and Polymerization Mechanisms

From the polyindene yield results measured with the precipitation method at different doses, the radiation chemical yield of polymerization G_p_ can be determined using [11]:

G_p_ = 9.648 × 10^6^ Q (q U m)^−1^ (2)

where G_p_ is monomer molecules/100 eV transformed into the polymer, Q is the polymer yield expressed in g, q is the mass of the irradiated monomer (in g), U is the dose in kGy and m is the monomer’s molecular weight (i.e., 116.16 Dalton for indene).

**Figure 5:**
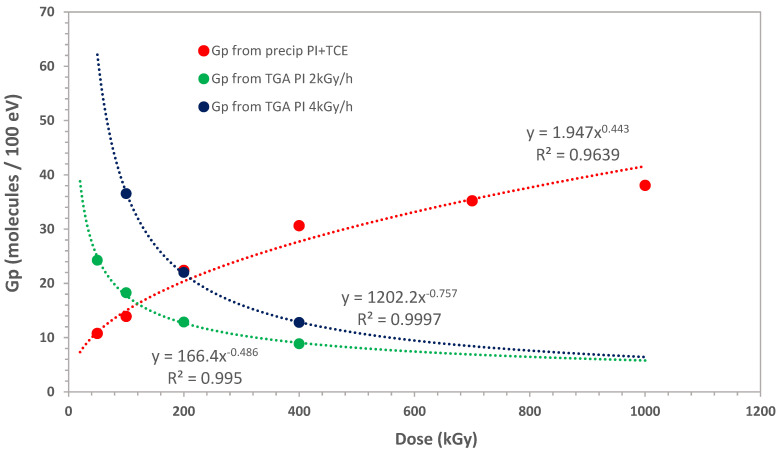
The radiation chemical yield G_p_ of indene polymerization sensitized with TCE follows a power law with positive exponent. In non-sensitized bulk polymerization of indene, the data can be fitted with a power law with negative exponent.

As shown in Figure 5, the radiation chemical yield experimental data can be fitted by a power law having the form of:

G_p_ = A U^−ϕ^ (3)

when the indene monomer is polymerized in bulk, using the experimental data of the previous work [1]. At 2 kGy/h the factor A = 166.4 and the exponent ϕ = 0.486, while at 4 kGy/h A = 1200 and ϕ = 0.757. The meaning of Equation (2) could be interpreted in terms of energy efficiency. At a given dose rate, the radiation chemical yield is maximized at a low dose and vanishingly small at very high doses.

It is noteworthy that Equation (3) has the same form of the equation linking the G_p_ with the dose rate Ů, with K including different constants [11,12,13]:

G_p_ = K Ů^−0.5^ (4)

In other words, the radiation chemical yield for the polymer formation is inversely proportional to the square root of the dose rate (Equation (4)), and in our study using Equation (3) we have found that this type of power law also applies to the total dose, although the exponent ϕ deviates from the 0.5 value.

The experimental data relative to the radiation-induced polymerization of indene sensitized by TCE can be fitted by a power law (Figure 5). However, this time the exponent of the dose U is positive:

G_p_ = A U^+ϕ^ (5)

with A = 1.947 and the exponent ϕ = 0.443. In the sensitized indene polymerization, the trend of G_p_ is just opposite to that observed in bulk and unsensitized polymerization.

It is evident that the presence of a sensitizer like TCE enhances the overall energetic efficiency of the process, especially at high doses.

The sensitizer acts essentially as an energy transfer agent, absorbing the high energy radiation in a better way than the monomer and then transferring the energy to the monomer. To exert this effect, the sensitizer in general is characterized by a higher sensitivity to high energy radiation with G_R_ (radiation chemical yield of radicals) values much higher with respect to the G_R_^(M)^, i.e., the radical radiation chemical yield of the monomer. For instance, Ivanov [11] reports G_R_ = 25, 37 and 44 radicals/100 eV respectively for CCl_4_, CHCl_3_ and CHBr_3_ used as sensitizers. These values should be compared with G_R_^(M)^ = 0.69 radicals/100 eV measured on pure styrene [14,15], a monomer similar to the chemical structure of indene [2]. TCE, with its structure Cl_2_CH-CHCl_2_, especially recalls the chemical structure of CHCl_3_ and should show G_R_ values similar to those of chloroform. Furthermore, the sensitization effect is not only a matter of a higher concentration of free radicals and hence easier polymerization initiation. The sensitizer could also address a parallel mechanism involving cationic polymerization, because the radiolysis of haloalkanes used as sensitizers generates certain ionic species which are also able to promote the cationic mechanism in parallel to the free radical, as already noticed by earlier investigators [14]. The overall effect of the sensitizer is translated in practice into a better utilization of the high energy radiation, leading to higher polymer yields, especially at higher doses. At lower doses, the effect in G_p_ values appears less evident, as can be observed in Figure 5.

**Scheme 1:**
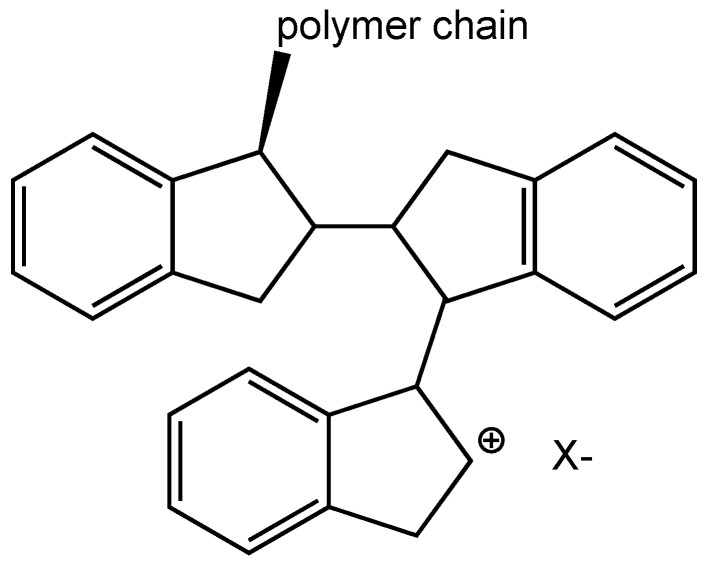
Interaction of the end groups of polyindene with HCl, yielding a polyindenyl cation [2].

Regarding the cationic mechanism which should be active in parallel to the free radical mechanism, and which contributes either to the enhancement of the polymerization kinetics and the polymer yield, it is necessary to make some considerations, starting from the radiolysis of pure halocarbons. While the radiolysis of CCl_4_ yields Cl_2_ (G = 0.75) together with other products but no HCl, the radiolysis of CHCl_3_, CH_2_Cl_2_, n-C_3_H_6_-Cl and Cl_2_C=CHCl produces HCl with G ≈ 5 molecules/100 eV together with other products but without any Cl_2_ [15]. Similar radiation chemical yield values for HCl production were recently observed in the radiolysis of 10 different chloroalkanes [16]. According to Milinchuk and Tupikov’s data [15], TCE represents an *unicum* with a behavior intermediate between CCl_4_ and the other chloroalkanes considered here, yielding simultaneously HCl (G = 7.2) and Cl_2_ (G = 2.7). From one side, chlorine is an electrophilic agent and a free radical source. depending on the conditions, and it may act either as initiator or as suppressant of the polymer chain growth. HCl is a known co-catalyst in cationic polymerization, and it plays a key role in stabilizing the growing polyindene chain through the complexation of the polyindene end groups [2] as shown in Scheme 1. Thus, the in situ HCl formation during the radiolysis of TCE may address the cationic mechanism of indene polymerization through said end groups’ complexation. Nonetheless, HCl could also act as a suppressant of polymer chain growth. Indeed, the overall action of Cl_2_ and HCl contributes to the production of oligomeric polyindenes with chlorinated end groups in a process called telomerization [11,12,13], as will be shown in the next sections.

Furthermore, studies on the radiolysis of TCE in hydrocarbons, like for instance cyclohexane [17], have revealed that the radiation chemical yield of HCl is dramatically enhanced G = 21.6 with the formation of trichloroethane G = 118, trichloroethyl radicals and numerous other chlorinated hydrocarbons, which can be generically represented as R-Cl.

According to Mehnert [12], in the irradiation of alkyl chlorides, the electron produced in the primary ion pair is converted to an unreactive chloride ion by dissociative electron capture leading to a radical:

R-Cl → R-Cl^+•^ + e_s_^−^ (6)

e_s_^−^ + R-Cl → Cl^−^ + R• (7)

while the alkyl chloride cation transfers its charge to the monomer M, initiating cationic polymerization [12,13]:

R-Cl^+•^ + M → M^+^ (8)

M^+^ + M → MM^+^ (9)

The cationic polymerization is further sustained by the relatively high dielectric constant of TCE (ε = 8.50 and μ = 1.32 Debye [18]) while indene ε ≈ 2.5. Thus, the above scheme proposed by Mehnert [12] explains the simultaneous free radical and cationic mechanism initiation. The chloride ion of reaction (7), being a nucleophile, can prematurely terminate the growing polymer chain through a cationic mechanism.

As deeply discussed in a previous work [2], the experimental evidence of the cationic polymerization of indene can be detected visually and spectrophotometrically. The polyindenyl cation gives a blood-red color to the solution, and such color is quite stable for a long time. Furthermore, the polyindenyl cation is characterized by an absorption band at 521 nm [2]. The solutions irradiated at 400, 700 and 1000 kGy are characterized by the blood-red color. The polyindenes, once precipitated with ethanol excess from the irradiated solutions, present a reddish color which persists even after drying. The KBr pellets of these polyindenes examined with the spectrophotometer show a broad but distinctive peak at 521 nm. Thus, the cationic polymerization mechanism was certainly in action in parallel with the free radical mechanism during the irradiation of the indene solutions in the presence of TCE.

### 3.3. Molecular Weight and Chemical Structure of Polyindene Obtained in Sensitized Radiation-Induced Polymerization

It is well known that the radiation-induced polymerization in the presence of sensitizers represented by chlorinated hydrocarbons is characterized by high initiation rates but also by high termination rates because of the high concentration of free radicals [11,12,13]. This condition leads to high polymer yields characterized by low molecular weight. Such a process is also known as telomerization [11,12,13].

The polyindene samples isolated with the precipitation method were subjected to X-ray fluorescence (XRF) analysis for the determination of the chlorine content. From these data, the elemental composition of the samples produced at the selected doses were determined with the basic and plausible assumption that the end groups of the polyindene chains are composed by chlorine atoms. This assumption is made on the basis of the radiolysis products of TCE, which involve mainly HCl and Cl_2_ (when radiolysed as pure liquid) [15].

The XRF analysis shows that the chlorine content in polyindene increases linearly with the dose. This fact is reasonable, since at higher doses there is more polymer yield but with shorter chains, as indeed is shown in Table 2.

An alternative and indirect way to estimate the molecular weight (M_n_) of polyindene is through the determination of the glass transition (T_g_) as shown in a previous work [2]. Using the equation proposed by Hahn and Hillmyer [19]:

Mn = −1.4747 × 10^5^ (T_g_ − 206.6)^−1^ (10)

the T_g_ of the polyindene samples was determined by DSC at a heating rate of 10 °C/min under N_2_ flow. Table 2 shows a summary of the T_g_ results and the corresponding molecular weight calculated with Equation (10). Indeed, in these polyindene samples obtained by radiolysis in the presence of the TCE sensitizer, the T_g_ was found at considerably lower temperatures than in the case of polyindenes synthesized by bulk radiolysis or by chemical initiations [2].

For instance, polyindene produced by radiolysis in bulk shows a T_g_ = 176.5 °C and Mn ≈ 5000 Da, while polyindene produced by cationic polymerization has a T_g_ = 205 °C and Mn ≈ 90,000 Da. Table 3 shows that all polyindenes produced in presence of TCE are characterized by T_g_ values between 152 °C and 120 °C, with the lowest T_g_ values at the higher doses. This fact, is translated into a confirmation of lower molecular weight for the polyindenes obtained in presence of the sensitizer and a good correlation with the molecular weight measured from the chlorine content.

Particularly interesting is the fact that the lowest molecular weight values are found on the samples prepared at higher doses. This is fully in line with the theory that predicts lower molecular weights of polymers at high doses, despite the higher mass yield.

**Figure 6:**
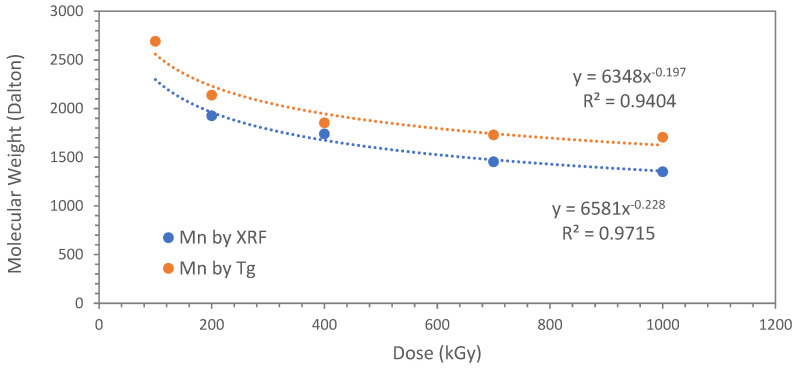
Molecular weight (M_n_) of the polyindene samples as determined from the chlorine content (XRF analysis) or from the T_g_ (glass transition by DSC analysis).

Despite the fact that the molecular weights derived from chlorine content (XRF analysis) and those estimated from the T_g_ (DSC analysis) are not numerically coincident, they follow exactly the same trend as shown in Figure 6, and the experimental values can be fitted by very similar power law equations with good R^2^ values.

Thus, fitting the molecular weight data with dose in the case of the T_g_ derived values:

M_n_(T_g_) = 6348 U^−0.197^ (11)

while for the M_n_ derived from XRF and chlorine end groups:

M_n_(Cl) = 6581 U^−0.228^ (12)

The latter two equations are very similar, and the most reliable should be considered Equation (12) which is linked to the direct measurement of the chlorine end groups and hence the true molecular weight. The other equation (Equation (11)), obtained through a completely different approach and based on the T_g_ of the polyindenes, represents a confirmation of the validity of Equation (12).

Based on these results, it appears immediately evident that the telomerization phenomenon [11] generated by the presence of the TCE sensitizer with high initiation and termination rates and high chain transfer reactions leads to polyindenes with relatively low molecular weights, in the range of 1350–2000 Da in comparison with 5000 Da measured on polyindenes that were radiation-polymerized in bulk without additives [1,2]. Furthermore, an interesting property of the polyindenes produced in presence of TCE regards the fact that higher polymer yields (achieved at higher doses) correspond with the lowest molecular weights and vice versa.

### 3.4. FT–IR Spectroscopy of the Polyindenes Obtained by Sensitized Radiation-Induced Polymerization

**Figure 7:**
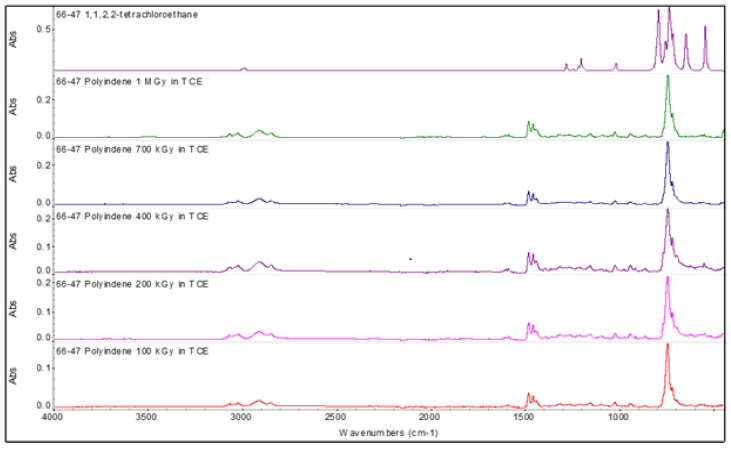
FT–IR spectra from top to bottom: TCE sensitizer, polyindene obtained at 1000, 700, 400, 200 and 100 kGy respectively.

The FT–IR spectra of polyindenes obtained either with γ irradiation of the indene monomer or with cationic or thermal initiations were already discussed in the previous works [1,2]. As shown in Figure 7 the polyindene FT–IR spectrum is dominated by a very strong absorption band at 746 cm^−1^ due to the out of plane wagging of four adjacent aromatic C-H groups of the indene aromatic ring [1,2]. This band is accompanied by another band at 718 cm^−1^ due to the indene CH_2_ rocking [1,2]. It was noticed that the latter band is better defined and well resolved in the polyindene obtained by radiation-induced polymerization of indene monomer in bulk and interpreted in terms of the high regularity of the resulting chemical structure of the polymer [1]. On the other hand, for the polyindene synthesized through a pure cationic mechanism, the band at 718 cm^−1^ appears poorly resolved and at the limit even appears as a simple shoulder on the main band at 746 cm^−1^. Figure 7 shows that this is also the case for all polyindenes synthesized in the present work using TCE as sensitizer; the band at 718 cm^−1^ is indeed poorly resolved. This experimental fact sustains the idea that the presence of TCE has favored the cationic mechanism (in parallel with the unavoidable free radical mechanism), leading to polyindene samples with chemical structures which also report the signature of such a mechanism on the infrared spectra. After all, it is well known that the chlorinated sensitizers also favor the cationic mechanism in radiation-induced polymerization [11,12,13,14].

The infrared spectrum of 1,1,2,2-tetrachloroethane is shown in Figure 7, and it is dominated by the C-Cl stretching bands at 795, 740, 716, 649 and 551 cm^−1^ [20,21]. It is a quite unfortunate situation that two of these main TCE infrared bands result nearly coincident with the 746 and 718 cm^−1^ main bands of polyindene. This precludes any possible attempt to detect the end groups of the polyindene samples obtained in the presence of the TCE sensitizer. In fact, apart from these two bands that if present are certainly buried by the polyindene vibrations, all the other C-Cl bands of TCE are not detectable at all in the polyindene spectra of the present work.

## 4. Conclusions

From the polyindene yield measured either with TGA or with polymer precipitation (gravimetry), the kinetic rate constant of TCE sensitized radiation-induced polymerization of indene was found at 3.11 × 10^−6^ mol L^−1^s^−1^ at a dose rate of 3 kGy/h, about one order of magnitude higher than the rate constant of radiation-polymerized indene in bulk without any additive.

In terms of the radiation chemical yield G_p_ determined according to Equation (2), the experimental data can be fitted with power laws. In TCE sensitized radiation-induced polymerization, G_p_ grows with the dose, while in the absence of the sensitizer G_p_ drops with the dose. This means that the sensitizer permits a higher efficiency in the utilization of the high energy radiation in conducting the polymerization with respect to the condition in the absence of a sensitizer.

Furthermore, the sensitizer is able to initiate the monomer polymerization according either with the free radical or with the cationic mechanism. Indeed, the radiolysed indene solutions show the presence of the polyindenyl cation at 521 nm in the electronic absorption spectra and evidences of the cationic polymerization in the final chemical structure of the polyindene (see Section 3.4 for a discussion).

The presence of TCE sensitizer implies a high initiation rate for the indene monomer, but also high chain transfer and termination rates. Thus, the molecular weight of polyindenes produced by the sensitized radiation-induced polymerization was found to be 1300–2000 Da, considerably lower than the polyindene molecular weight obtained with radiation polymerization in bulk (5000 Da). The lowest molecular weight was that of the polyindene sample produced at 1000 kGy, in coincidence with the maximum polymer yield in terms of polymer mass recovered after the polymerization.

## Figures and Tables

**Table 1:** Polyindene yield as determined either by TGA method and by precipitation method.

	100 kGy	200 kGy	400 kGy	700 kGy	1000 kGy
% by TGA	5.35	10.19	18.44	33.12	44.54
% Precipitation	1.67	5.39	14.75	29.67	45.79

**Table 2:** Chlorine content of polyindene samples obtained in the presence of TCE with the resulting molecular weight.

	200 kGy	400 kGy	700 kGy	1000 kGy
% Carbon	89.83	89.49	89.78	88.45
% Hydrogen	6.70	6.67	6.62	6.60
% Chlorine (*)	3.47	3.84	4.60	4.95
Molecular Weight (M_n_ Dalton)	1925	1739	1453	1350

(*) Chlorine was determined by XRF, carbon and hydrogen knowing that indane is the repeating unit; M_n_ calculated assuming chlorine atoms as end groups.

**Table 3:** Comparison of polyindene molecular weight by XRF and by T_g_.

kGy	M_n_ by XRF (Dalton)	M_n_ by Tg (Dalton)	Tg (°C)
100	n.d.	2691	151.8
200	1925	2137	137.6
400	1739	1853	127.0
700	1453	1729	121.3
1000	1350	1705	120.1

## Data Availability

This work will be available on the Researchgate website at the authors’ page (see: https://www.researchgate.net/profile/Franco-Cataldo/stats accessed on 1 May 2025). Further inquiries can be directed to the corresponding author.
